# Supplementary material for: Pharmacokinetics of Haloperidol in Critically Ill Patients: Is There an Association with Inflammation?
Source: Pharmaceutics. 2022 Feb 28;14(3):549. doi: 10.3390/pharmaceutics14030549 (PMC8950491; doi:10.3390/pharmaceutics14030549)
Supplement: Supplementary file 1 [file pharmaceutics-14-00549-s001.zip › pharmaceutics-1586240 - supple/MDPI haloperidol Supplementary Material/Supplementary Materials File A-The Structural model and covariate analysis equation.pdf]

Structural model

The logarithmic transformed concentration data were best described by a one-compartment model with an additive residual error. Inter-individual variability (IIV) was included on CL.

$CL_{i,j} = CL_{pop} * exp\ (\eta_i)$

Covariate analysis

Continuous covariates were tested using the following equation:

Eq. 1: $CL_{i,} = [CL_{pop} * (COV/median\ COV)^{\Theta cov}] * exp\ (\eta_i)$

Categorical covariates were implemented as shown in equation 2:

Eq. 2: $CL_{i,} = CL_{pop} * (\Theta_{cat1})^{FLAG1} * (\Theta_{cat2})^{FLAG2} * exp\ (\eta_i)$

The $\Theta_{cat1,2...}$ is the typical value under different categorical conditions The FLAG (1,2…) are first set to 0, when certain categorical covariate appears, the FLAG is set to 1.
