# Supplementary material for: Pharmacokinetics of Haloperidol in Critically Ill Patients: Is There an Association with Inflammation?
Source: Pharmaceutics. 2022 Feb 28;14(3):549. doi: 10.3390/pharmaceutics14030549 (PMC8950491; doi:10.3390/pharmaceutics14030549)
Supplement: Supplementary file 1 [file pharmaceutics-14-00549-s001.zip › pharmaceutics-1586240 - supple/MDPI haloperidol Supplementary Material/Supplementary Materials File C-METC ethical approval of the research.pdf]

Erasmus MC
Universitair Medisch Centrum Rotterdam

Medische Ethische Toetsings Commissie
Erasmus MC
www.erasmusmc.nl/commissies/metc/

Mw.dr. N.G.M. Hunfeld
Afd. Intensive Care
Kamer Na-119
Erasmus MC

Doorkiesnummer +31 10 7033625/34428
Faxnummer
Kamernummer Z 514
E-mail metc@erasmusmc.nl
Ons kenmerk MdV/hl/215531
Datum 21 juli 2014

Betreft: MEC-2014-264 Positief besluit NL48875.078.14 v05

Postadres
Postbus 2040
3000 CA Rotterdam

Bezoekadres
's Gravendijkwal 230
3015 CE Rotterdam

Titel onderzoek:
'Pharmacologic Evaluation of Intravenous Haloperidol for the Treatment of Delirium in Critically Ill Adults'

Verrichter: Erasmus MC
Singlecenter

Geachte mevrouw Hunfeld,

De Medisch Ethische Toetsings Commissie Erasmus MC (METC) heeft het bovengenoemde onderzoeksvoorstel ontvangen op 6 mei 2014.

Besluit
De commissie heeft het onderzoeksvoorstel in het kader van de Wet medisch-wetenschappelijk onderzoek met mensen (WMO) besproken in de 952^{e} vergadering d.d. 20 mei 2014. Naar aanleiding van deze toetsing heeft de commissie u enkele vragen c.q. opmerkingen voorgelegd. De vragen en opmerkingen zijn naar tevredenheid beantwoord.

De commissie oordeelt **positief** over de uitvoering van het onderzoek in het volgende centrum:

- Erasmus MC te Rotterdam

Alvorens met de uitvoering van het onderzoek in het Erasmus MC gestart mag worden, dient u in het bezit te zijn van toestemming voor de uitvoering van het onderzoek door het afdelingshoofd.

Voorzitters
Prof.dr. H.W. Tilanus
Prof.dr. H.J. Metselaar

Vice voorzitter
Dr. C.M. Zwaan

Secretarissen
Mw. mr. C.P. Bron-van Vliet
Mw.dr.ir. M.M.C. de Vries-Velraeds
Mw.ing. W.C.M. Tielemans
Drs. H. van der Baan (a.i.)

Secretaressen
Mw. A. de Jong
Mw. S. Sneevliet
Mw.drs. H.L. Lammers-Assems

Adm. medewerker
Mw. A.E. van Huuksloot

Het secretariaat is geopend van maandag tot en met vrijdag van 08.30 tot 17.00 uur

WMO proefpersonenverzekering
De commissie verleent ontheffing van de verplichting een verzekering af te sluiten, die de door het onderzoek veroorzaakte schade van de proefpersoon dekt. De commissie is van oordeel dat aan dit onderzoek voor de proefpersoon naar zijn aard hooguit verwaarloosbare/geen risico's zijn verbonden.

Melden
De volgende gegevens moeten aan de commissie worden gemeld:

- Startdatum (datum waarop de eerste proefpersoon is geïncludeerd)
- Einde inclusie (datum waarop de laatste proefpersoon is geïncludeerd)

601077

Erasmus MC
Universitair Medisch Centrum Rotterdam

- Einddatum (datum waarop de laatste meting bij de laatste proefpersoon heeft plaatsgevonden)

Voorleggen ter beoordeling
De volgende documenten moeten aan de commissie ter beoordeling worden voorgelegd:

- Onverwachte en ernstige bijwerkingen via portal ToetsingOnline
- Amendementen en/of addenda
- Formulier Jaarlijkse voortgangsrapportage, voor het eerst één jaar na dit besluit
- Publicaties en eindrapport

BROK cursus
Voorts wijst de commissie de onderzoekers erop dat, conform landelijke afspraken, klinisch onderzoekers verplicht zijn de 'Basiscursus regelgeving en organisatie van klinisch onderzoek' (BROK) te doorlopen en het bijbehorende certificaat te behalen. De commissie gaat ervan uit dat, indien dit nu nog niet het geval is, de klinisch onderzoekers van deze studie maximaal 6 maanden na aanvang van de studie aan deze verplichting voldaan hebben. Voor informatie over de BROK-cursusdata kunt u contact opnemen met het Congresbureau, intern tel.nr. 43584.

Beroep
Tot slot wijs ik u erop, dat op grond van artikel 23 van de Wet medisch-wetenschappelijk onderzoek met mensen juncto artikel 7: 1 van de Algemene wet bestuursrecht, degene wiens belang rechtstreeks bij dit besluit is betrokken daartegen binnen zes weken na de dag waarop dit besluit bekend is gemaakt, een administratief beroepschrift kan indienen bij de Centrale Commissie Mensgebonden Onderzoek. Een dergelijk administratief beroepschrift dient u te adresseren aan: CCMO, Postbus 16302, 2500 BH Den Haag.

Geldigheid besluit
De commissie heeft de bevoegdheid haar positieve besluit in te trekken als vaststaat dat de uitvoering van het onderzoek ernstig tekortschiet. Het positieve besluit van de commissie verliest geldigheid als met de uitvoering van het onderzoek niet is begonnen binnen één jaar nadat dit besluit is genomen.

Met vriendelijke groet,
namens de Medisch Ethische Toetsings Commissie Erasmus MC,

Mw.dr.ir. M.M.C. de Vries-Velraeds
Secretaris

601?187

Erasmus MC
Universitair Medisch Centrum Rotterdam

Beoordeelde documenten

- Aanbiedingsbrief en akkoordverklaring hoofdonderzoeker, mw.dr. N.G.M. Hunfeld, en afdelingshoofd, dr. D.A.M.P.J. Gommers, zoals ontvangen d.d. 6 mei 2014;
- ABR-formulier NL48875.078.14 versie 05 d.d. 7 juli 2014;
- Addendum bij ABR-formulier, zoals ontvangen d.d. 6 mei 2014;
- Onderzoeksprotocol, versie 3.0 d.d. juli 2014;
- Delirium protocol, versie 2 d.d. 18 februari 2014;
- SPC Haldol, 10 mg tabletten, 2 mg/ml druppels, 5 mg/ml oplossing voor injectie, herzien d.d. 13 juni 2013;
- Patiënteninformatie- en toestemmingsformulier patiënt, versie 3.0 d.d. 15 juli 2014;
- Patiënteninformatie- en toestemmingsformulier patiënt na wettelijk vertegenwoordiger, versie 3.0 d.d. 15 juli 2014;
- Informatie- en toestemmingsformulier wettelijk vertegenwoordiger, versie 3.0 d.d. 15 juli 2014;
- Informatie waaruit blijkt dat ontheffing van de verzekeringsplicht kan worden verleend;
- Verzekeringscertificaat d.d. 16 maart 2012 inzake aansprakelijkheidsverzekering Erasmus MC;
- Curriculum vitae onafhankelijke arts prof.dr. T. van Gelder, getekend d.d. 25 april 2014;
- Curriculum vitae hoofdonderzoeker mw.dr. N.G.M. Hunfeld, getekend d.d. 25 april 2014; en
- Monitoringplan 'verwaarloosbaar risico' inclusief bijlage A, getekend d.d. 25 april 2014.

Samenstelling METC Erasmus MC:

Dr. K.M. Akkerhuis	Cardioloog
Dr. P.A.W. te Boekhorst	Internist-hematoloog
Prof.dr. A.H.J. Danser	Klinisch farmacoloog
Dr.ir. M.T. Hilhorst	Ethicus
Prof.dr. H.J. Metselaar (voorzitter)	Internist / MDL-arts
Dr. R. Timman	Methodoloog
Mw.mr. C.F.B Verhagen-van Weerden	Gezondheidsjurist
Mw.dr.ir. M.M.C. de Vries-Velraeds	Secretaris
Prof.dr. A.G. Vulto	Ziekenhuisapotheker/klinisch farmacoloog
Mw.dr. S.N. de Wildt	Kinderarts
Mw.drs. S.E. Zijlstra	Invalshoek proefpersoon / Extern lid

Bijlage Certificaat d.d. 16 maart 2012 inzake aansprakelijkheidsverzekering Erasmus MC

Cc. **Digitale verzending:**
Dr. D.A.M.P.J. Gommers
CCMO

6017187

MEC-2014-264
singlecenter, geldt uitsluitend voor het Erasmus MC

CNA

CERTIFICAAT VAN VERZEKERING
MEDISCHE AANSPRAKELIJKHEID

Polisnummer	:	NLAMT00081

Ondergetekende verzekert:

Verzekeringnemer	:	Erasmus Universitair Medisch Centrum<br>'s-Gravendijkwal 230<br>3015 CE Rotterdam
Verzekerde hoedanigheid	:	Universitair Medisch Centrum in de breedste zin van het woord
Verzekerd bedrag	:	Zaak- en letselschade<br>€ 2.500.000,- per aanspraak en<br>€ 10.000.000,- per jaar<br><br>Verlies van documenten (sublimiet)<br>€ 2.500.000,- per aanspraak en<br>€ 10.000.000,- per jaar<br><br>Opzichtschade (sublimiet)<br>€ 2.500.000,- per aanspraak en<br>€ 10.000.000,- per jaar<br><br>Zuivere vermogensschade (sublimiet)<br>€ 2.500.000,- per aanspraak en<br>€ 10.000.000,- per jaar
Verzekeringsperiode	:	Deze verzekering is gesloten aanvangende 1 januari 2012 voor een periode van 36 maanden, nader omschreven in de clausule verzekeringsperiode
Dekkingsgebied	:	Wereldwijd, exclusief USA/Canada

Verder overeenkomstig alle voorwaarden en condities als omschreven in de polis met Polisnummer NLAMT00081

Amsterdam, 16 maart 2012

CNA Insurance Company Ltd.

CNA

21 juli 2014

Mw.dr.Ir. M.M.C. de Vries-Velraeds
secretaris Medisch Ethische Toetsingscommissie
Erasmus MC
Rotterdam
