# Supplementary material for: Pharmacokinetics of Haloperidol in Critically Ill Patients: Is There an Association with Inflammation?
Source: Pharmaceutics. 2022 Feb 28;14(3):549. doi: 10.3390/pharmaceutics14030549 (PMC8950491; doi:10.3390/pharmaceutics14030549)
Supplement: Supplementary file 1 [file pharmaceutics-14-00549-s001.zip › pharmaceutics-1586240 - supple/MDPI haloperidol Supplementary Material/Supplementary Materials File D-Informed consent statement.pdf]

Informatiebrief
Onderzoek naar de spiegels in het bloed van haloperidol bij patiënten met een delier op de Intensive Care afdeling

Geachte heer/mevrouw,

Uw partner of familielid is opgenomen op de Intensive Care afdeling van het Erasmus MC. Op dit moment doen wij op de Intensive Care onderzoek naar de werking van een medicijn (haloperidol) dat wordt gegeven bij een delier. Een delier komt veel voor op de Intensive Care. Omdat een delier ook bij uw partner of familielid zou kunnen ontstaan, vragen wij uw aandacht voor dit medisch-wetenschappelijk onderzoek. Uw partner of familielid is op dit moment niet in staat te beslissen over deelname aan dit onderzoek. Omdat u de wettelijke vertegenwoordiger bent, vragen wij u te beslissen over zijn/haar. Mocht u toestemming geven en uw partner/familielid krijgt een delier, dan zullen we bij hem/haar het onderzoek uitvoeren. Als hij/zij geen delier ontwikkelt, gebeurt er niets. Voordat u de beslissing neemt, is het belangrijk om meer te weten over het onderzoek. Lees deze informatiebrief rustig door. Lees ook de algemene brochure. Daar staat veel informatie over medisch-wetenschappelijk onderzoek in.

Heeft u na het lezen van de informatie nog vragen? Dan kunt u terecht bij de onderzoeker. De contactgegevens staan onderaan de brief.

1. Wat is het doel van dit onderzoek?

Iemand die is opgenomen op de Intensive Care heeft vaak meerdere lichamelijke problemen. Hierdoor kan een delier ontstaan. Een delier komt op de Intensive Care afdeling voor bij 24-82% van de patiënten. Het wordt veroorzaakt door disfunctioneren van de hersenen. Patiënten zijn vaak plotseling erg verward, hebben aandachtsproblemen en andere symptomen. Het is belangrijk om een delier goed en snel te behandelen, omdat een delier ervoor kan zorgen dat iemand langer opgenomen is in het ziekenhuis en de kans op sterfte verhoogd is.
Patiënten die op de Intensive Care een delier ontwikkelen worden behandeld met het medicijn haloperidol. Ondanks dat dit middel al jaren wordt gebruikt bij de behandeling van een delier, weten we niet goed hoe dit medicijn zich in het bloed gedraagt bij patiënten op de Intensive Care. In dit onderzoek wordt gekeken naar de hoeveelheid haloperidol (spiegel) in het bloed en het effect op de symptomen van delier.

2. Hoe wordt het onderzoek uitgevoerd?

Als degene die u vertegenwoordigt een delier ontwikkelt, zal hij/zij worden behandeld met het medicijn haloperidol. Deze behandeling verandert niet als hij of zij mee doet aan het onderzoek. Het afbreken van haloperidol wordt gedaan door de lever. Om te meten hoe snel de lever dit doet, worden er op dag 1 van het onderzoek twee extra buizen bloed afgenomen van 3 ml. Hierbij wordt in het DNA gekeken. Het DNA zal worden bewaard voor eventueel toekomstig onderzoek naar delieren. De tweede dag van het onderzoek worden er vier buisjes bloed van 1 ml afgenomen. Daarna wordt dagelijks 1 buisje bloed afgenomen van 1 ml, zolang uw partner of familielid het geneesmiddel haloperidol toegediend krijgt, met een maximum van 5 dagen. Nadat het bloed is afgenomen wordt de hoeveel haloperidol in het bloed uit het buisje bepaald. In totaal wordt er dus maximaal 11 ml bloed afgenomen. Uw partner/familielid heeft een infuuslijn in een bloedvat waardoor dagelijks bloed afgenomen wordt voor de behandeling. Door deze lijn zal ook het bloed worden afgenomen dat nodig is voor het onderzoek. Hij of zij hoeft voor dit onderzoek dus niet extra worden geprikt. In onderstaande afbeelding kunt u nogmaals zien, hoe het onderzoek precies wordt uitgevoerd.

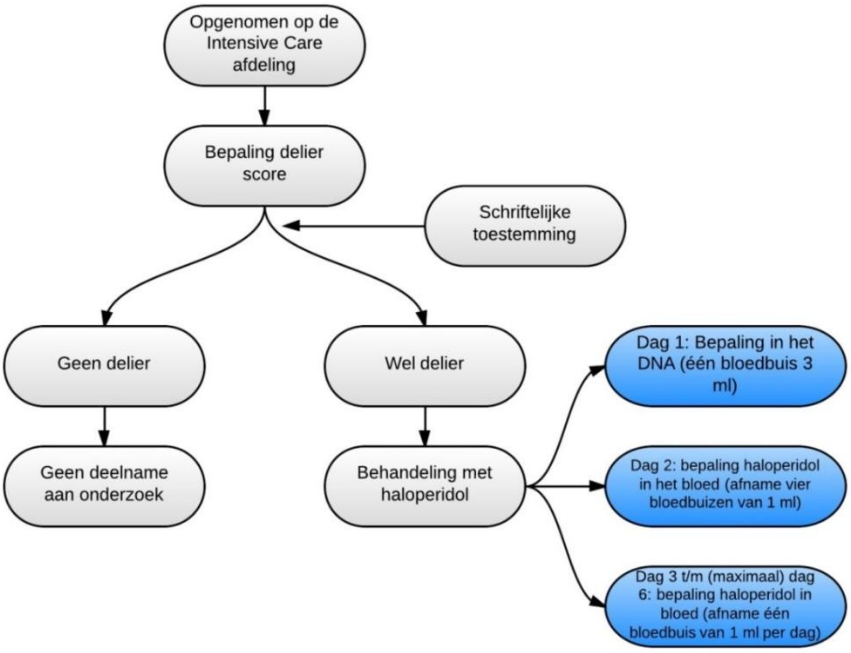

3. Wat zijn de mogelijke voor- en nadelen van deelname aan het onderzoek?

Uw partner of familielid heeft zelf geen voordeel van deelname aan dit onderzoek. In de toekomst kan het onderzoek wel nuttige informatie opleveren voor andere patiënten. Een nadeel van het onderzoek is dat er maximaal 9 extra buizen bloed worden afgenomen, maar dit gebeurt uit een infuus dat uw partner/familielid al heeft. Uw partner of familielid wordt voor dit onderzoek niet extra geprikt.

4. Wat gebeurt er als u geen toestemming geeft voor deelname aan dit onderzoek?
U beslist of uw partner of familielid meedoet aan het onderzoek. Deelname is vrijwillig. Als u besluit dat hij of zij niet mee doet, hoeft u verder niets te doen. U hoeft niets te tekenen. U hoeft ook niet te zeggen waarom u niet wilt dat uw partner of familielid mee doet. Hij of zij krijgt gewoon de behandeling die hij/zij anders ook zou krijgen. Als u besluit dat uw partner of familielid wel meedoet, kunt u zich altijd bedenken en uw toestemming intrekken. Ook tijdens het onderzoek. Mocht uw partner of familielid tijdens het onderzoek weer in staat zijn te beslissen over deelname, kan hij/zij altijd besluiten het onderzoek te stoppen.

5. Is uw partner of familielid verzekerd wanneer hij/zij aan het onderzoek meedoet?
De toetsende commissie heeft ontheffing verleend van de verplichting een verzekering af te sluiten voor de deelnemers aan dit onderzoek, omdat zij van mening is dat dit onderzoek weinig of geen risico met zich meebrengt.

6. Wat gebeurt er met de gegevens?
In de algemene brochure is uitgelegd dat de onderzoeker gegevens over uw partner of familielid verzamelt en deze vertrouwelijk behandelt. Dit betekent dat een aantal personen de medische status en de gegevens van het onderzoek mogen inzien. Deze personen mogen de gegevens gebruiken voor dit onderzoek, maar zij mogen deze gegevens alleen bekend maken zonder daarbij de naam of andere persoonlijke gegevens te vermelden. De identiteit van uw partner of familielid blijft dus altijd geheim. De onderzoeker bewaart de gegevens met een code. Dit betekent dat op de studie-documenten in plaats van de naam enkel een letter-cijfercode staat. Alleen de onderzoeker houdt een lijst bij waarop staat welke letter- cijfercode bij welke naam hoort. Normaal gesproken heeft alleen de behandelend arts en zijn/ haar team inzage in de gegevens van uw partner/familielid. Als u toestemming geeft voor deelname aan de studie krijgen meer mensen inzage in de medische gegevens en studiegegevens. De personen die inzage kunnen krijgen in de gegevens van uw partner/familielid zijn:

- de medewerkers van het onderzoeksteam,
- de leden van de toetsingscommissie die de studie heeft goedgekeurd,
- de bevoegde medewerkers van de Inspectie voor de Gezondheidszorg

Na de studie worden de gecodeerde gegevens gedurende 15 jaar bewaard. Dit is nodig om alles goed te kunnen controleren. Na de studie wordt het bloed en DNA van uw partner of familielid in gecodeerde vorm gedurende 15 jaar bewaard. Wij willen dit

materiaal graag gebruiken voor andere onderzoeken die worden uitgevoerd naar delieren. Deze onderzoeken hebben dus eenzelfde doel als het onderzoek waarvoor u nu wordt gevraagd. Het is dus niet zo dat uw materiaal ook zal worden gebruikt voor onderzoek naar een geheel andere aandoening of een heel ander probleem. Vanzelfsprekend blijft de vertrouwelijkheid die we hierboven hebben beschreven altijd gelden. Mogelijk willen we uw partner/familielid in de toekomst opnieuw benaderen voor vervolgonderzoek. Op het toestemmingsformulier kunt u aangeven of u dat goed vindt.

7. Welke medisch-ethische toetsingscommissie heeft dit onderzoek goedgekeurd?
De Medisch Ethische Toetsingscommissie (METC) van het Erasmus MC heeft dit onderzoek goedgekeurd. Meer informatie over de goedkeuring vindt u in de algemene brochure.

8. Wilt u verder nog iets weten?
Mocht u na het lezen van deze brief nog vragen hebben over het onderzoek, kunt u contact met ons opnemen. Onze gegevens staan in de bijlage.
Indien u twijfelt over deelname kunt u een onafhankelijke arts raadplegen, die zelf niet bij het onderzoek betrokken is, maar die wel deskundig is op het gebied van dit onderzoek en uw ziekte. Ook als u vragen over de studie heeft die u liever niet aan de onderzoekers stelt, kunt u contact opnemen met de onafhankelijke arts. Zijn gegevens vindt u in de bijlage.

Indien u na zorgvuldige overweging besluit dat uw partner of familielid deel gaat nemen aan dit wetenschappelijk onderzoek, dan vragen we u om samen met de onderzoeker het toestemmingsformulier te ondertekenen en te dateren.

Met vriendelijke groet,
Het onderzoeksteam

Bijlagen

1. Contactgegevens
2. Toestemmingsformulier
3. Algemene Brochure Medisch-Wetenschappelijk onderzoek bij mensen

Bijlage 1 - Contact gegevens

1. Onze gegevens

Voor vragen of opmerkingen over het onderzoek kunt u op werkdagen tussen 9:00 en 17:00 contact opnemen met de onderzoeker.

Dr. Nicole Hunfeld, ziekenhuis apotheker Intensive Care.
Telefoonnummer: 06-39016233
E-mail: N.Hunfeld@erasmusmc.nl

Dr. Mathieu van der Jagt, Intensivist/Neuroloog, afdeling Intensive Care
Tel: 010-7030478

2. Onafhankelijk arts

Prof. dr. T. van Gelder, internist-nefroloog.
Telefoonnummer: 010- 7033202
E-mail: t.vangelder@erasmusmc.nl

3. Klachtencommissie

Mocht u tijdens het onderzoek of na afloop klachten hebben over de uitvoering van het onderzoek, kunt u contact opnemen met de klachtencommissie. U kunt een brief sturen naar:

Erasmus MC
T.a.v. secretaris klachtencommissie
Postbus 2040
3000 CA Rotterdam

Bijlage 2 Toestemmingsformulier

Onderzoek naar de spiegels in het bloed van haloperidol bij patiënten met een delier op de Intensive Care afdeling.

Ik ben gevraagd om toestemming te geven voor de volgende persoon, zodat hij meedoet aan dit medisch-wetenschappelijk onderzoek:

Naam proefpersoon: Geboortedatum: __ / ___ / _____

Ik heb de informatiebrief voor de wettelijk vertegenwoordiger gelezen. Ik kon aanvullende vragen stellen. Deze vragen zijn naar tevredenheid beantwoord. Ik heb voldoende tijd gehad om te beslissen of deze persoon meedoet.
Ik weet dat deelname helemaal vrijwillig is. Ik weet dat ik op ieder moment kan beslissen dat deze persoon toch niet meedoet. Daarvoor hoef ik geen reden te geven.

Ik geef toestemming om de specialist(en), die deze persoon behandelt, te vertellen dat hij/zij meedoet aan dit onderzoek.

Ik weet dat sommige mensen de gegevens van deze persoon kunnen zien. Die mensen staan vermeld in de algemene brochure.

Ik geef toestemming om de gegevens te gebruiken voor de doelen die in de informatiebrief staan.

Ik geef toestemming om de gegevens van degene die ik vertegenwoordig nog maximaal 15 jaar na afloop van de studie te bewaren.

Ik geef wel/geen* toestemming om DNA van mijn partner/familielid nog maximaal 15 jaar na afloop van dit onderzoek te bewaren voor toekomstig onderzoek met eenzelfde onderzoeksdoel.

Ik geef wel/geen* toestemming om mijn partner/familielid in de toekomst opnieuw te benaderen voor vervolgonderzoek.

Ik vind het goed dat deze persoon meedoet aan dit onderzoek.

Naam wettelijk vertegenwoordiger:

Relatie tot proefpersoon:

Handtekening : Datum : __ / __ / __

Ik verklaar hierbij dat ik deze persoon/personen volledig heb geïnformeerd over het genoemde onderzoek.

Als er tijdens het onderzoek informatie bekend wordt die de toestemming van de proefpersoon zou kunnen beïnvloeden, dan breng ik hem/haar daarvan tijdig op de hoogte.

Naam onderzoeker :

Handtekening : Datum: __ / __ / __

---

* Doorhalen wat niet van toepassing is.
