# Supplementary material for: Pharmacokinetics of Haloperidol in Critically Ill Patients: Is There an Association with Inflammation?
Source: Pharmaceutics. 2022 Feb 28;14(3):549. doi: 10.3390/pharmaceutics14030549 (PMC8950491; doi:10.3390/pharmaceutics14030549)
Supplement: Supplementary file 1 [file pharmaceutics-14-00549-s001.zip › pharmaceutics-1586240 - supple/MDPI haloperidol Supplementary Material/Supplementary Materials File E-Validation of the method for haloperidol determination.pdf]

Analysevoorschrift

Midazolam en Haloperidol in plasma (UPLC-MS/MS Waters Xevo TQ-S micro 1)

Autorisatie:

**hoedanigheid**	**naam**
auteur<br>beoordelaar<br>autorisator	W. ten Bosch-Dijksman<br>S. Bahmany<br>B.C.P. Koch

Verzendlijst:

**locatie (toegewezen in KIS)**	**papieren versie (logboek/werkmap/ruimte)**	**beheerder papieren versie**
laboratorium	logboek midazolam en haloperidol: 1x	Kwaliteitsadviseur laboratorium

INHOUDSOPGAVE

INHOUDSOPGAVE 2
1 ONDERWERP / DOELSTELLING 3
2 TOEPASSINGSGEBIED 3
3 DEFINITIES EN TERMEN 4
4 VOORZORGEN EN VEILIGHEID 4
4.1 Werkplek 4
4.2 Materiaal 4
4.3 Reagentia 4
4.4 Afvalverwerking 4
5 UITVOERING 5
5.1 Benodigdheden 5
5.1.1 Apparatuur en hulpmiddelen 5
5.1.2 Grondstoffen 5
5.1.3 Reagentia 5
5.1.4 Standaarden en Controles 6
5.1.4.1 Stockstandaarden 6
5.1.4.2 Werkstandaarden 6
5.1.4.3 Interne standaard (ISTD) 7
5.1.4.4 Controle (QC) 8
5.1.4.5 Etikettering stock- en werkstandaarden 9
5.1.5 Patiëntmateriaal 9
5.2 Voorbereidingen 9
5.3 Werkwijze / Activiteiten 9
5.3.1 Monstervoorbehandeling 9
5.3.2 Analyse 9
6 RAPPORTAGE 10
6.1 Beoordeling van de resultaten 10
6.2 Registratie van de resultaten 11
6.3 Archivering 11
7 VERANTWOORDELIJKEN EN VERANTWOORDELIJKHEDEN 11
8 SPECIFICATIES METHODE N.A.V. VALIDATIE 11
8.1 Detectiegrenzen & precisie 11
8.2 Houdbaarheid extracten 12
9 NASLAG 12
9.1 Verwante documenten Apotheek 12
9.2 Referenties 12
10 BIJLAGEN 13

1 ONDERWERP / DOELSTELLING

Dit analysevoorschrift beschrijft de werkwijze bij de kwantitatieve analyse van onderstaande componenten in plasma m.b.v. de UPLC-MS/MS.

1. Midazolam
2. α-Hydroxy-midazolam
3. Hydroxy-midazolam glucuronide
4. Haloperidol

Midazolam wordt gebruikt als premedicatie, inleiding van de narcose, sedatie bij ingrepen onder lokale anesthesie en bij slaapstoornissen. De halfwaardetijd van de serumspiegel bedraagt ongeveer 2 uur.
Haloperidol wordt vaak op de IC in combinatie gegeven met midazolam, vanwege een studie is de vraag of haloperidol toegevoegd kon worden aan de bepaling midazolam.

2 TOEPASSINGSGEBIED

Dit analysevoorschrift is van toepassing voor alle analisten.
De analyse is bedoeld voor de kwantitatieve bepaling van midazolam en haloperidol in plasma, ten behoeve van TDM.
Het bereik van de bepaling, de bijbehorende therapeutische ranges en de toxische concentraties van de verschillende componenten staan in tabel 2.1 weergegeven.
Na een chromatografische scheiding tussen onderstaande componenten, de interne standaard (midazolam-d4 en haloperidol-d4) en storende componenten van de biologische matrix met behulp van UPLC worden bovengenoemde componenten gekwantificeerd door middel van MS/MS-detectie.

Tabel 2.1 Therapeutische en toxische ranges

Component	Ther. Range (µg/l, dal)	Toxische Conc. (µg/l, dal)	Meetbereik (µg/l)
Midazolam	80-250 (Als hypnoticum) 1000-2400 (Als sedativum)	1000 (Totaal)*	2 – 2400
α-Hydroxy-midazolam	2/3 t.o.v. midazolam spiegel		3 - 2300
Hydroxy-midazolam glucuronide	10 % t.o.v. midazolam spiegel		10 – 3000
Haloperidol	5-15	20-25 Kind 10-20	0,5 - 20

* De toxische concentratie betreft de som van de 3 componenten, rekening houdend met hun werkzaamheid (t.o.v. Midazolam).

3 DEFINITIES EN TERMEN

ESI	Electrospray Ionization (ionisatietechniek voor LC-MS)
FA	Formic Acid (mierenzuur)
ISTD	Interne standaard
LIS	Laboratorium Ziekenhuis Informatie Systeem
LC-MS/MS	Liquid Chromatography Tandem Massa Spectrometrie
LLOQ	Lower Limit of Quantitation
LOD	Limit of Detection
LOQ	Limit of Quantitation
MRM	Multiple Reaction Monitoring
QC	Quality Control (controlestandaard)
RT	Retentietijd
SST	System Suitability
TDM	Therapeutic Drug Monitoring
ULOQ	Upper Limit of Quantitation
UPLC	Ultra Performance Liquid Chromatography

4 VOORZORGEN EN VEILIGHEID

4.1 Werkplek

De met patiëntmateriaal in aanraking gekomen oppervlakken worden schoongemaakt met water en zeep en vervolgens afgenomen met een vezelarme doek gedrenkt in een alcoholisch desinfectans. Het oppervlak wordt aan de lucht gedroogd voordat wordt begonnen met nieuwe werkzaamheden.

4.2 Materiaal

Behandel patiëntmateriaal als potentieel infectieus materiaal, zie werkinstructie docnr. 8.5.23 (omgaan met biologisch materiaal op het lab).

4.3 Reagentia

Acetonitril:	Licht ontvlambaar, schadelijk
Methanol:	Licht ontvlambaar, giftig
Mierenzuur:	Bijtend
Humaan serum:	zie procedure Vernietigen van geneesmiddelen en overige afvalstromen, doc.nr. 7.3.1.3

4.4 Afvalverwerking

Afval wordt verwerkt en afgevoerd volgens de procedure ‘Vernietigen van geneesmiddelen en overige afvalstromen’.
Verwerk oplossingen die methanol of acetonitril (kunnen) bevatten, waaronder eluens en (verdunningen van) standaardoplossingen, als vloeibaar chemisch afval (halogeen arme oplosmiddelen Hoogcal).
Verwerk (mogelijk) biologisch besmet afval, waaronder buizen met patiëntmateriaal, pipetpunten e.d., als ziekenhuisafval, zie procedure Vernietigen van geneesmiddelen en overige afvalstromen, docnr. 7.3.1.3

5 UITVOERING

5.1 Benodigdheden

5.1.1 Apparatuur en hulpmiddelen

Autosampler vials:	Snap ring vial, 32 x 11,6mm, VWR
Autosampler vial caps:	Snap cap, preslit, Waters Chromatography
Centrifuge:	Eppendorf microcentrifuge
Detectie:	ESI in positieve mode, MRM
Eluens:	Zie § 5.1.3
Eppendorf-cupjes:	1,5 ml
Kolom:	2.1 x 100 mm Waters Acquity UPLC BEH C_{18} 1.7 μm, cat no. 186002352
LC:	Waters Acquity UPLC (binary solvent manager, sample manager, sample organizer, column manager)
MS:	Waters Xevo TQ-S mirco
Rollerbank:	Thermo Spiramix
Software:	MassLynx, TargetLynx
Ultrasoonbad:	Branson 3510

5.1.2 Grondstoffen

Ammoniumacetaat	Fluka, cat no 17836
α-hydroxy-Midazolam	Lipomed, MID-330-FB-1LM
Haloperidol-d4	Sigma-Aldrich, H-002
Hydroxy-Midazolam glucuronide	AlsaChim, cat no C1243
Hydroxy-Midazolam glucuronide	TRC, cat no H948430
Methanol	Biosolve, LC-MS, catno. 13687801
Midazolam	Lipomed, MID-111-FB-1LM
Midazolam-d4	Lipomed, MID-1767-MA-1LM
Mierenzuur	Biosolve ULC/MS, catno. 06914131
Water	Millipore, MilliQ® Advantage A10® System
QC 1 en 2 Midazolam	Recipe ClinChek® Serum Controls, for Benzodiazepines, Level I en II, art. No. MS6082
QC 3 Midazolam	Chromsystems MassCheck® Benzodiazepines 1 plasma control level II, art. No. 0239
Std 1 en 2 Haloperidol	Chromsystems MassCheck® Neuroleptics 1 EXTENDED plasma controls, art. No. 0210/XT
QC L, M en H Haloperidol	Chromsystems 3PLUS1® Multilevel Plasma Calibrator set Neuroleptics 1 EXTENDED, art. No. 92028/XT
Blanco Humaan Plasma	Vers bevroren plasma, Sanquin Bloedbank. Afhaaladres: Hemostase lab Erasmus MC

5.1.3 Reagentia

ISTD oplossing	Zie ook § 5.1.4.3
Standaard 1 t/m 4	Zie ook § 5.1.4.2
Controlemonsters	Zie ook § 5.1.4.4
0,5% mierenzuur	Voeg 0.5 ml mierenzuur toe aan 100 ml MilliQ water
Eluensconcentraat	2M Ammoniumacetaat in mierenzuur: Los 7,7 gram ammoniumacetaat op in 50 ml mierenzuur.
Eluens A	2mM Ammoniumacetaat in LC-MS water

	Voeg 1 ml eluensconcentraat toe aan 1 liter MilliQ water toe en homogeniseer (maximaal 1 maand houdbaar).
Eluens B	2mM Ammoniumacetaat in LC-MS methanol<br>Voeg 1 ml eluensconcentraat toe aan 1 literfles LC-MS methanol en homogeniseer (maximaal 3 maanden houdbaar).
Weak Wash	90:10 (v/v) MilliQ Water : Methanol. Neem 900 ml MilliQ water en voeg 100 ml MeOH toe en meng (maximaal 1 maand houdbaar).
Strong Wash	LC-MS Methanol (maximaal 3 maanden houdbaar).

5.1.4 Standaarden en Controles

5.1.4.1 Stockstandaarden

Van de grondstoffen worden afzonderlijke stockoplossingen gemaakt voor de standaarden en controles. De stockstandaard voor de component OH-midazolam glucuronide wordt bereid door nauwkeurig 10 mg af te wegen en over te brengen in een maatkolf van 100 ml (zie tabel 5.1). Vul aan tot de maatstreep met methanol. Homogeniseer de oplossing en plaats de stockstandaard 30 minuten in het ultrasoonbad. De concentratie van de stockstandaard is ca. 100 mg/l.

Voor de stockstandaarden van midazolam en OH-midazolam worden ampullen gebruikt die kant en klaar in oplossing geleverd worden door de desbetreffende leverancier. De concentratie van deze ampullen is 1 mg/ml (= 1000 mg/l). Van deze ampullen worden afzonderlijke stockoplossingen gemaakt door een hoeveelheid (zie tabel 5.1) van de ampul over te pipetteren met een positive displacement pipet in een maatkolf en vul aan tot de maatstreep met methanol. Homogeniseer de oplossing. Zie tabel 5.1 voor de voorbeeld concentraties.

Tabel 5.1 Stockstandaarden

Stockstandaard Component	Inweeg (mg)	Maatkolf (ml)	Conc. +/- (mg/l)
Midazolam	1000 µl Ampul	50	20
OH-Midazolam	1000 µl Ampul	50	20
OH-Midazolam-glucuronide	10	100	100

De houdbaarheid van de stockstandaarden bedraagt 2 jaar bij -20 °C (kanaal 78).

5.1.4.2 Werkstandaarden

Vanuit de stockstandaarden worden de werkstandaarden bereid met uitzondering van de hydroxymidazolam glucuronide, hiervoor wordt een tussenstockstandaard vers bereid.

De tussenstockstandaard van hydroxymidazolam glucuronide wordt bereid door 1 ml stockstandaard hydroxymidazolam glucuronide over te brengen in een maatkolf van 10 ml en vul aan tot de maatstreep met methanol. De concentratie van de stockstandaard is ongeveer 10 mg/l. Deze tussenstockstandaard wordt voor iedere standaard werkoplossing vers bereid.

De werkstandaarden worden bereid volgens het schema in tabel 5.2 in een maatkolf van 10 ml, vul aan tot de maatstreep met plasma. Vul bij voorkeur de QC's dezelfde dag uit door middel van 100 µl m.b.v. een repeteerpipet over te brengen in afzonderlijke epjes.
De houdbaarheid van de werkstandaarden bedraagt 1 jaar bij -80°C (kanaal 83).

Tabel 5.2 Standaarden midazolam

	Standaard 1	Standaard 2	Standaard 3
Aantal µ stock Midazolam	10	50	125
Aantal µ stock OH-midazolam	10	50	125
Aantal µ tussenstock OH-midazolam glucuronide	60	150	300
Volume maatkolf	10	10	10
Eindconcentratie (µg/l) Midazolam	≈ 20	≈ 100	≈ 250
Eindconcentratie (µg/l) OH-Midazolam	≈ 20	≈ 100	≈ 250
Eindconcentratie (µg/l) OH-Midazolam glucuronide	≈ 60	≈ 150	≈ 300

Haal voor het bereiden van de standaarden haloperidol de in §5.1.2 vermelde gevriesdroogde Chromsystems set uit de vriezer en voeg 1 ml toe aan de Chromsystems *(0210/XT)*. Laat vervolgens 15 minuten bij kamertemperatuur staan en leg ze daarna nog 15 minuten op de rollerbank. Vul de standaarden vervolgens uit door met een repeteerpipet (met een pipetpunt van 2,5 ml) 100µl over te brengen in epjes. De houdbaarheid van de gereconstitueerde standaarden bedraagt 3 maanden bij -80°C.

Benoem de uitgevulde standaarden als volgt;
- Midazolam "Std1" en "Std2"
- Haloperidol "Std1" en "Std2"

Tevens voorzien van lotnummer en expiratie datum.

Zie voor de exacte gegevens van de in gebruik zijnde standaarden het logboek 'Beheer standaarden Bio-analyse', LG-L045.

5.1.4.3 Interne standaard (ISTD)

Voor de interne stockstandaard worden ampullen gebruikt die kant en klaar in oplossing geleverd worden door de desbetreffende leverancier. De concentratie van deze ampullen is voor midazolam-d4 1000 µg/ml en voor haloperidol-d4 100 µg/ml.

Van deze ampullen worden afzonderlijke stockoplossingen gemaakt door 1000 µl van de ampul midazolam-d4 over te brengen in een maatkolf van 100 ml en voor haloperidol-d4 1000 µl van de ampul over te brengen in een maatkolf van 10 ml. Vul vervolgens aan tot de maatstreep met methanol en homogeniseer de oplossingen. De concentratie van de stockstandaard midazolam-d4 en haloperidol-d4 is 10 mg/l. De houdbaarheid van de stockstandaarden bedraagt 2 jaar bij -20 °C (kanaal 78).

De interne standaard werkoplossing wordt bereid door 200 µl van de stockstandaard midazolam-d4 en 100 µl stockstandaard haloperidol-d4 over te brengen in een maatkolf van 100 ml. Vul vervolgens aan tot de maatstreep met methanol en homogeniseer de oplossing. De eindconcentratie is 20 µg/l voor midazolam-d4 en 10 µg/l haloperidol-d4 . De oplossing is 1 jaar houdbaar bij 2-8 °C (kanaal 57).

5.1.4.4 Controle (QC)

Bij deze analyse zijn 4 controlemonsters (QC's) in gebruik. In tabel 5.2 staat weergegeven welke component in welke controle zit.

Tabel 5.2: Samenstelling QC's

Component	Waters Target-methode	Recipe QC 1 en 2	Chromsystems QC 3	Eigen Bereiding QC	Chroms. QC L, M en H
Midazolam	A	X	X		
OH-midazolam	B	X	X		
OH-midazolam glucuronide	C			X	
Haloperidol	D				X

Haal voor bereiding van de controles de in tabel 5.2 vermelde gevriesdroogde Recipe en/of Chromsystems set uit de koelkast/vriezer en voeg zoals op de flacon vermelde hoeveelheid MilliQ water toe aan de desbetreffende controle. De diverse sets kunnen afzonderlijk van elkaar in gebruik genomen worden. Laat vervolgens 15 minuten bij kamertemperatuur staan en leg ze daarna nog 15 minuten op de rollerbank.
Vul de controles vervolgens uit door met een repeteerpipet (met een pipetpunt van 2,5 ml) 100µl over te brengen in epjes. De houdbaarheid van de gereconstitueerde controles van Recipe en Chromsystems bedraagt 3 maanden bij -80°C.

Er wordt een eigenbereiding controle gebruikt ter controle van OH-midazolam glucuronide. De tussenstockstandaard van hydroxymidazolam glucuronide wordt bereid door 1 ml stockstandaard *(let op, een aparte stock t.b.v. de standaarden en controles)* hydroxymidazolam glucuronide over te brengen in een maatkolf van 10 ml en vul aan tot de maatstreep met methanol. De concentratie van de stockstandaard is ongeveer 10 mg/l. Deze tussenstockstandaard wordt voor iedere standaard werkoplossing vers bereid.
De controles worden bereid volgens het schema in tabel 5.3 in een maatkolf van 10 ml, vul aan tot de maatstreep met plasma. Vul bij voorkeur de QC's dezelfde dag uit door middel van 100 µl m.b.v. een repeteerpipet over te brengen in afzonderlijke epjes.
De houdbaarheid van de werkstandaarden bedraagt 1 jaar bij -80°C (kanaal 83).

Tabel 5.3 QC OH-midazolam glucuronide

	Controle Laag	Controle Middel	Controle Hoog
Aantal µ tussenstock	100	200	350
Volume maatkolf	10	10	10
Eindconcentratie (µg/l)	≈ 100	≈ 200	≈ 350

Benoem de uitgevulde controles als volgt;

- Midazolam "QC1" en "QC2"
- Midazolam "QC3"
- Haloperidol "QC L", "QC M" en "QC H"

Tevens voorzien van lotnummer en expiratie datum.

Zie voor de exacte gegevens van de in gebruik zijnde controlemonsters het logboek 'Interne QC LCMS', LG-L066-3.

5.1.4.5 Etikettering stock- en werkstandaarden

Gebruik voor alle standaarden (m.u.v. de uitgevulde QC's en werkstandaarden in eppendorf eppjes) de Lassie2 software. Vermeld op het etiket minimaal: De naam van de standaard, de naam van de desbetreffende doelcomponent(en) met bijbehorende concentratie(s), bereider, bereidingsdatum, expiratiedatum en oplosmiddel.
Zie voor codering van de in eppendorfepjes standaarden en/of controles de bovenstaande §5.1.4.2 en §5.1.4.4.

5.1.5 Patiëntmateriaal

De monsterafname geschiedt extern. Het monstermateriaal wordt na ontvangst gedurende 6 minuten bij 3000 toeren per minuut gecentrifugeerd.
Het monster wordt in de koelkast bewaard tot analyse (zie weekschema, 7.5.1 bijlage 5).
Na analyse wordt het monster gedurende 1 week in de koelkast bewaard.

5.2 Voorbereidingen

Haal de blanco, de werkstandaarden in duplo, interne standaard werkoplossing, 0.5% mierenzuur, controles en de patiëntmonsters volgens een LIS-werklijst uit de koelkast of vriezer.
Laat deze minimaal 30 minuten staan om op kamertemperatuur te komen.
Zet voldoende lege eppendorfepjes klaar voor de blanco en de patiëntmonsters.

5.3 Werkwijze / Activiteiten

5.3.1 Monstervoorbehandeling

- Pipetteer in alle klaargezette cupjes 100 μl van respectievelijk blanco plasma en de patiëntmonsters.
- Voeg aan ieder cupje 50 μl 0.5% mierenzuur in MilliQ-water toe met een repeteerpipet.
- Voeg vervolgens aan ieder cupje 600 μl interne standaard oplossing toe met een repeteerpipet en sluit de dopjes.
- Meng de oplossingen 10 seconden op de vortex en centrifugeer vervolgens 5 minuten bij 14680 rpm.
- Breng het supernatant over in een autosampler vial.
- Injecteer het supernatant in de volgorde volgens de voorbeeld samplelist (bijlage 6).
- Injecteer de oplossingen volgens de instellingen in § 5.3.2.

5.3.2 Analyse

Zet het systeem klaar volgens de checklist (zie bijlage 2).

Controleer de LC-MS-opstelling:

Kolom	2.1 x 100 mm Waters Acquity UPLC BEH C18 1.7 μm (Positie 2)
Flow	0.4 ml/min
Eluens A	Algemeen eluens A
Eluens B	Algemeen eluens B
Weak wash	Algemene wash
Strong wash	Algemene strong wash
Initiële druk	ca. 650 bar
Gradiënt	(zie ook bijlage 3)

	Tijd(min)	*eluens A*	*eluens B*	*Curve*
	Initieel	70%	30%	Init.
	1.9	0%	100%	6
	2.5	0%	100%	1
	3.2	70%	30%	1
Kolomtemperatuur	60 °C			
Injectortemperatuur	10 °C			
Injectieloop	20 µl			
Injectie volume	3 µl			
Datapath	C:\Masslynx Projects			
Masslynx project	Midazolam_Haloperidol.PRO			
Inlet file	Midazolam_Haloperidol (zie ook bijlage 3)			
MS Tune file	Midazolam_Haloperidol.ipr (zie ook bijlage 4)			
MS file	Midazolam_Haloperidol.exp (zie ook bijlage 5)			
Targetlynx XS methode	Midazolam_Haloperidol.mdb (zie ook bijlage 8)			

Maak een samplelist (zie bijlage 6, voorbeeld samplelist MassLynx), controleer of de druk stabiel is (Acquity software, Binary Solvent Manager, delta p < 10) en start de LC-MS zoals beschreven in de bedieningsinstructie LC-MS Waters Xevo TQ-S micro 1, docnr. 5.2.2.5.79.
Vergelijk het eerste chromatogram met het voorbeeldchromatogram (bijlage 7). Vul aan de hand van dit chromatogram de system suitability checks in op de verkorte werkinstructie (bijlage 1).

Indien na analyse het systeem niet handmatig uitgezet wordt (bijv. omdat er overnacht gedraaid wordt), dient achter de desbetreffende samplelist een spoelfile gedraaid te worden. Op deze manier wordt de kolomflow en kolomtemperatuur verlaagd naar resp. 0 ml/min en kamertemperatuur. Open hiervoor samplelist in het desbetreffende project "*Spoelfile*" en start de eerste en enige regel van de bijbehorende acquisition sequence.

Verwerk na afloop van de totale samplelist de resultaten zoals beschreven in de bedieningsinstructie.

6 RAPPORTAGE

6.1 Beoordeling van de resultaten

Kalibratie:

- De afwijking van de piekoppervlakte van de interne standaard mag in de afzonderlijke chromatogrammen maximaal 15% afwijken van het gemiddelde.
- Correlatiecoëfficient: r > 0.995; Determinatiecoëfficient: r^2 > 0.990.
- De duplowaarden verschillen maximaal 10% van elkaar.

Controles:

- De resultaten dienen te voldoen aan de eisen in Unity Real Time.

Patiëntmonsters:

- De integratie van de pieken is juist uitgevoerd.
- De afwijking van de piekoppervlakte van de interne standaard mag in de afzonderlijke chromatogrammen maximaal 15% afwijken van het gemiddelde.
- De retentietijden van de pieken komen overeen met die van de standaarden.

6.2 Registratie van de resultaten

Voer de resultaten van de controles in BioRad Unity Real Time in (zie bedieningsinstructie 5.2.2.5.51).
Voer de waardes van de patiëntmonsters in het LIS in aan de hand van de LIS-werklijst.
Leg de ruwe data klaar voor autorisatie door een bevoegd analist en de laboratoriumapotheker.

6.3 Archivering

De resultaten (ruwe data print-outs) worden gearchiveerd en gedurende 1 jaar bewaard. De resultaten (ruwe data elektronisch) worden permanent bewaard.

7 VERANTWOORDELIJKEN EN VERANTWOORDELIJKHEDEN

verantwoordelijken	verantwoordelijkheden
Analist	- voorbereiding, uitvoering en afronding van de analyse<br>- beoordeling kalibratie en controles<br>- invoer resultaten in het LIS
Bevoegd analist	- autorisatie ingevoerde gegevens<br>- interpreteren en rapporteren van afwijkende resultaten
Ziekenhuisapotheker laboratorium	- uitbrengen behandeladvies aan behandelaars<br>- eindverantwoordelijke voor analyseresultaten, interpretatie en rapportage aan kliniek

8 SPECIFICATIES METHODE N.A.V. VALIDATIE

8.1 Detectiegrenzen & precisie

De detectiegrenzen staan in onderstaande tabel weergegeven (tabel 8.1).

Tabel 8.1: Detectiegrenzen en precisie

	**LOD (µg /l)**	**LLOQ (µg /l)**	**ULOQ (µg /l)**	**Juistheid <15 (%)**		
				**L**	**M**	**H**
Midazolam	1.2	2	2400	-7.4	-1.8	-3.9
α-OH-midazolam	1.2	3	2300	4.0	-3.1	-6.8
OH-midazolam-glucuronide	1.42	10	3000	-4.8	-6.2	-3.6
Haloperidol	0.12	0.5	31*	-9.9	-6.7	-8.7

	**Herhaalbaarheid <15 (%)**			**Reproduceerbaarheid <15 (%)**			**Meetonzekerheid <15 (%)**		
	**L**	**M**	**L**	**L**	**M**	**H**	**L**	**M**	**H**
Midazolam	1.5	4.1	1.4	2.4	2.0	3.7	7.8	2.7	5.4
α-OH-midazolam	2.5	0.9	2.6	3.3	3.3	4.5	5.2	4.5	8.2
OH-midazolam-glucuronide	2.7	1.5	2.5	4.1	4.1	4.2	6.3	7.5	5.5
Haloperidol	1.2	1.1	2.2	2.8	2.4	2.7	10.3	7.1	9.1

*De ULOQ is aangepast naar de hoogste concentratie van de gemeten QC's voor de juistheid van haloperidol.

8.2 Houdbaarheid extracten

De houdbaarheid van de aangeprikte vials is maximaal 24 uur.

9 NASLAG

9.1 Verwante documenten Apotheek

Bedieningsinstructie:	UPLC-MS/MS (Waters Xevo TQ-S micro 1), docnr. 5.2.2.5.79
Bedieningsinstructie:	Biorad Unity Time QC programma, docnr. 5.2.2.5.51
Procedure:	Vernietigen van geneesmiddelen en overige afvalstromen, docnr. 7.3.1.3
Procedure:	Klinisch farmaceutische en toxicologische analyses', docnr. 7.5.1 (bijlage 7 'Overzicht Bio-bepalingen')
Procedure:	Archiveren van documenten, docnr. 7.7.3.6
Werkinstructie:	Etiketten printen m.b.v. Lassie 2, docnr. 8.1.2.1
Werkinstructie:	Handelingen bij uitvoeren van analyses op het biolab, docnr. 8.5.19
Werkinstructie:	Omgaan met biologisch materiaal op het laboratorium, docnr. 8.5.23
Werkinstructie:	Bereiden, registreren en controleren van standaarden binnen het biologisch laboratorium, docnr. 8.5.50

9.2 Referenties

[1] Waters Mass Lynx 4.1; Getting Started Guide (71500113203/Revision A)
[2] Waters Acquity UPLC system; Quick Start Guide (71500082503/Revision B)
[3] Waters Micromass Quattro Premier XE Mass Spectrometer; Operator's Guide (71500082503/Revision B)
[4] ten Bosch-Dijksman W.; Validatie midazolam en haloperidol in plasma m.b.v. UPLC-MS/MS (Waters Quattro Premier) Apotheek Erasmus MC 2015 (LG-An036_2)
[5] ten Bosch-Dijksman W.; Partiele validatie midazolam en haloperidol in plasma m.b.v. UPLC-MS/MS (Waters Xevo TQ-S micro 1) Apotheek Erasmus MC 2018 (LG-An036_3)

10 BIJLAGEN

1. Verkorte werkinstructie
2. Checklist
3. Masslynx Inlet file
4. Masslynx MS Tune file
5. Masslynx MS file
6. Voorbeeld sample-list
7. Voorbeeld chromatogram
8. Quanlynx methode
